# Adipose Tissue Dysfunction and Impaired Metabolic Health in Human Obesity: A Matter of Oxygen?

**DOI:** 10.3389/fendo.2015.00055

**Published:** 2015-04-24

**Authors:** Gijs H. Goossens, Ellen E. Blaak

**Affiliations:** ^1^Department of Human Biology, NUTRIM School of Nutrition and Translational Research in Metabolism, Maastricht University Medical Centre, Maastricht, Netherlands

**Keywords:** adipose tissue, oxygen tension, obesity, hypoxia, hyperoxia, blood flow, metabolism, inflammation

## Introduction

**Figure 1:**
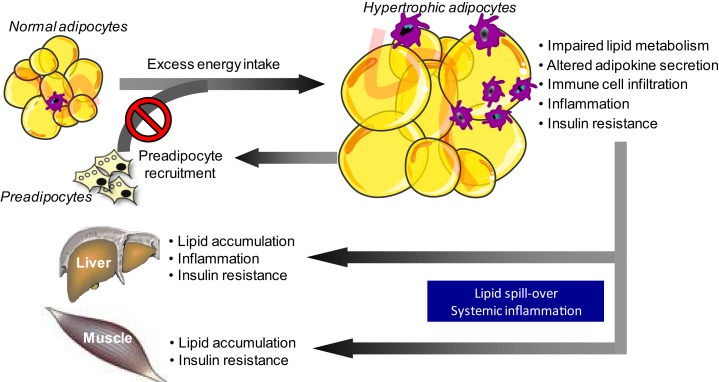
**Adipose tissue dysfunction in obesity is related to impaired metabolic health**. A long-term positive energy balance, leading to body weight gain, will increase adipocyte size. Adipocyte hypertrophy in obesity is accompanied by disturbances in lipid metabolism and alterations in adipokine secretion, which a shift toward a pro-inflammatory phenotype. The secretion of pro-inflammatory factors, which also impair adipocyte differentiation, is further boosted by the infiltration of several adaptive and innate immune cells into the adipose tissue in obesity. Together, the impairments in lipid metabolism and the secretory function of adipose tissue not only induce insulin resistance locally within the tissue (via autocrine/paracrine effects) but also have detrimental effects at the whole-body level. The reason for this is that adipose tissue dysfunction in obesity is accompanied by lipid spillover in the circulation and subsequent lipid accumulation in non-adipose tissues (ectopic fat storage), and may contribute to systemic low-grade inflammation, thereby accelerating the development and progression of obesity-related insulin resistance and chronic diseases.

The number of studies in the field of adipose tissue biology has increased exponentially over the last decade. This shift in research focus is primarily driven by the tremendous increase in the prevalence of obesity and related chronic diseases, including cardiovascular disease and type 2 diabetes mellitus. Adipose tissue is a fascinating and complex organ, with marked effects on whole-body physiology. Intriguingly, expansion of adipose tissue does not necessarily translate into increased metabolic and cardiovascular disease risk. A proportion of obese individuals seems to be relatively protected against worsening of metabolic health (1), suggesting that adipose tissue dysfunction, rather than the amount of fat mass, may be a key factor in the pathophysiology of obesity-related metabolic and cardiovascular diseases (2–4). It is widely accepted that impairments in adipose tissue lipid metabolism, a decreased adipose tissue blood flow (ATBF) and an increased production of pro-inflammatory cytokines by hypertrophic adipocytes and infiltrating adaptive and innate immune cells are characteristics of dysfunctional adipose tissue in obesity (2, 5). These impairments not only induce insulin resistance locally in the adipose tissue but also have detrimental effects at the whole-body level, thereby affecting metabolic health. The reason for this is that adipose tissue dysfunction in obesity is accompanied by lipid spillover in the circulation and subsequent lipid accumulation in non-adipose tissues (ectopic fat storage), and may contribute to systemic low-grade inflammation, thereby accelerating the development and progression of obesity-related insulin resistance and chronic metabolic diseases (Figure 1) (2).

## Adipose Tissue Oxygen Tension in Human Obesity

Since adipose tissue dysfunction has been recognized as a key process in the pathophysiology of obesity-related disorders (2, 5), the number of studies aimed at identifying the trigger of adipose tissue dysfunction in obesity has increased substantially. A prevailing concept is that an insufficient amount of oxygen within adipose tissue, commonly referred to as “adipose tissue hypoxia,” may underlie adipose tissue dysfunction in obesity (6, 7).

### Adipose tissue hypoxia in obesity: The concept

It has been postulated that adipose tissue angiogenesis is insufficient to maintain normoxia in the entire fat depot during the progressive development of obesity (8). In other words, a reduced supply of oxygen to the tissue has been proposed to instigate adipose tissue dysfunction. Indeed, a lower expression of angiogenic genes (e.g., VEGF) and lower capillary density have been found in abdominal subcutaneous adipose tissue of obese as compared to lean individuals (9, 10). The net result of structural and functional properties of the adipose tissue vasculature determines tissue blood flow. A consistent observation made by our lab and others is that fasting and postprandial ATBF is decreased in obese insulin resistant versus lean insulin sensitive subjects (9, 11–13), indicating that oxygen delivery to adipose tissue is indeed impaired in obesity. We have recently demonstrated, for the first time, that both pharmacological (local administration of vasoactive agents into adipose tissue) and physiological (oral glucose drink) manipulation of ATBF induce concomitant alterations in adipose tissue oxygen partial pressure (AT PO_2_) in humans (9), indicating that adipose tissue oxygen supply indeed affects AT PO_2_. A second argument that has been put forward to develop the concept of adipose tissue hypoxia in obesity is that the diameter of hypertrophic adipocytes in obesity exceeds the normal diffusion distance of oxygen across tissues (100–200 μm) (14). However, in human adipose tissue, there seems to be only a very small proportion of adipocytes with a diameter >100 μm (9, 15, 16). Therefore, the significance of reduced oxygen diffusion from the capillaries to hypertrophic fat cells in obese humans can be questioned.

### Adipose tissue hypoxia in obesity: What is the evidence in rodents and humans?

Several rodent studies have been performed to investigate whether adipose tissue hypoxia is present in obesity. These experiments have shown that massive and rapid weight gain in *ob/ob*, KKAy, and dietary-induced obese mice was accompanied by increased expression of hypoxia-responsive genes, more hypoxic areas (assessed using pimonidazole hydrochloride) and lower PO_2_ in white adipose tissue (17–19). Thus, multiple lines of evidence suggest that AT PO_2_ is lower in rodent models of obesity as compared to lean control animals. Importantly, impaired angiogenesis might be a more important determinant of AT PO_2_ in animal models of obesity than in human obesity, since the rate and extent of fat mass gain is much higher in mouse models of obesity (20). Intriguingly, there is only marginal evidence to substantiate the view that a relative oxygen deficit is present in adipose tissue in human obesity. So far, three studies have addressed whether abdominal subcutaneous adipose tissue of obese humans is “hypoxic” (9, 10, 21), of which two have directly measured AT PO_2_ (9, 10). First, Pasarica and colleagues (10) have found that AT PO_2_ (measured using polarographic Clark electrodes) was significantly lower in overweight/obese versus lean subjects. In this study however, groups were not matched for age, gender, ethnicity, and the presence of type 2 diabetes, which may have influenced the results. In contrast, we have recently demonstrated, using continuous optochemical PO_2_ monitoring, that obese insulin resistant subjects had significantly higher AT PO_2_ compared to lean insulin sensitive individuals, matched for age, gender, and ethnicity, despite significantly lower ATBF in the obese (9). Finally, Hodson and co-workers (21) have recently assessed a metabolic signature of abdominal subcutaneous adipose tissue by arterio-venous difference methodology. If large areas of adipose tissue would be “hypoxic” in obesity, this is likely reflected by a switch to anaerobic metabolism, i.e., an increased secretion of lactate and pyruvate from adipose tissue into the venous blood draining adipose tissue. However, the authors did not find any evidence for a metabolic signature typical for adipose tissue hypoxia in obese humans. Currently, we are investigating for the first time whether diet-induced weight loss will alter AT PO_2_ in overweight and obese individuals. This study will provide further insight into AT PO_2_ in human obesity.

What could explain a higher AT PO_2_, if indeed present, in human obesity? Since AT PO_2_ is the result of the balance between oxygen supply (ATBF) and the metabolic rate of the tissue (20), it may be that adipose tissue oxygen consumption is substantially lower in obesity. Indeed, animal studies have shown that mitochondrial morphology is abnormal, that mitochondrial biogenesis and mass are reduced, and that oxygen consumption is lower in both white and brown adipose tissue of obese Zucker rats (22), *ob/ob* mice (23), *db/db*, and high-fat diet-fed mice (24). In line, microarray-based gene expression profiling has demonstrated that increases in fat mass were paralleled by progressive downregulation of metabolic pathways, including mitochondrial energy metabolism, and upregulation of inflammatory pathways in both visceral and abdominal subcutaneous adipose tissue in humans (25). In accordance, data from our lab and others seem to indicate that *in vivo* adipose tissue oxygen consumption is lower in obese than lean subjects (9, 21). More recently, it has been demonstrated that human adipocyte mitochondrial content and mitochondrial oxygen consumption by white adipocytes is lower in obese compared to lean subjects (26). Interestingly, the latter study found that both small and large white adipocytes of obese individuals have a lower metabolic rate compared to adipocytes derived from lean donors (26), suggesting that impaired mitochondrial oxygen consumption might be a primary factor that contributes to adipocyte hypertrophy. Taken together, there is substantial evidence that the metabolic rate of adipose tissue is lower in obese humans, which may underlie the higher AT PO_2_ that we have previously found in obese humans (9). Clearly, it is the balance between oxygen supply and consumption that determines AT PO_2_ (20).

## Consequences of Altered Oxygen Tension: A Matter of Severity, Duration, and Pattern?

Many *in vitro* studies have been performed to investigate whether oxygen tension is involved in the regulation of metabolic and inflammatory processes in adipose tissue, as extensively reviewed elsewhere (6, 20). Earlier studies examined the effects of acute, short-term (1–24 h) exposure to extremely low PO_2_ (usually 1% O_2_) as compared to a ~20-fold higher O_2_ concentration (ambient air, 21% O_2_) on the expression and/or secretion of key adipokines involved in inflammation and metabolism. Most of these studies have demonstrated that extremely low PO_2_ induces a pro-inflammatory response in *3T3-L1* adipocytes (18, 27–31), human adipocytes (32), stromal–vascular cells (33, 34), and macrophages (18, 35), although conflicting results have also been reported (36, 37). On the other hand, 95% O_2_ also increased pro-inflammatory gene expression and reactive oxygen species (ROS) content, and reduced glucose uptake in *3T3-L1* adipocytes (38). These *in vitro* studies should be interpreted with some caution, because these cells were *acutely* exposed to extremely low (or high) PO_2_. In addition, two independent laboratories, including ours, have recently provided evidence that human abdominal subcutaneous AT PO_2_ ranges between ~3 and 11% O_2_ (~23–84 mmHg) (9, 10). Therefore, cell culture experiments investigating the effects of *chronic* exposure to more *physiological* PO_2_ are urgently warranted.

Studies that have examined the relationship between *in vivo* AT PO_2_ and the inflammatory phenotype of adipose tissue in humans have yielded conflicting results, showing both positive (9) and inverse (10) correlations. Interestingly, human primary adipocytes have recently been exposed to physiological PO_2_ levels (5 versus 10 versus 21% O_2_) during differentiation (14 days) (39). Of note, 5 and 10% O_2_ reflect the mean AT PO_2_ values that we have previously found in lean insulin sensitive and obese insulin resistant individuals, respectively (9). The authors were able to demonstrate that exposure to 10% O_2_ increased adipocyte triacylglycerol (TAG) content and boosted the secretion rates of IL-6 and DPP-4 (39). Interestingly, DPP-4 is involved in cross talk between adipose tissue and skeletal muscle, and has been shown to inhibit skeletal muscle insulin signaling (40).

Based on the human data from our laboratory, showing increased AT PO_2_ in obese insulin resistant subjects, a positive correlation between AT PO_2_ and adipose tissue gene expression of several pro-inflammatory markers, and an inverse association between AT PO_2_ and peripheral insulin sensitivity (9), we questioned whether chronic hypoxia exposure would have beneficial effects on the adipose tissue inflammatory and metabolic phenotype. Therefore, we exposed 52-week-old C57Bl/6J mice to chronic hypoxia (8% O_2_) or normoxia (21% O_2_) for 21 days, after which adipose tissue and plasma were collected. Chronic hypoxia exposure improved adipose tissue function in these mice, evidenced by decreased adipocyte size, increased adipose tissue gene expression of mitochondrial function markers, and decreased adipose tissue macrophage infiltration and gene expression of inflammatory markers (41), which may contribute to improved insulin sensitivity. More recently, the same concept has been applied to humans. Interestingly, exposure to moderate hypoxia (15% O_2_) for 10 subsequent nights significantly increased whole-body insulin sensitivity in obese men (42). Although not commented upon by the authors, moderate hypoxia exposure also tended to reduce AT PO_2_ (42). Therefore, these findings may imply that the decreased AT PO_2_ after moderate hypoxia exposure for 10 consecutive nights has contributed or even driven the improved peripheral insulin sensitivity, as recently postulated (43). Importantly, obstructive sleep apnea syndrome (OSAS), which is characterized by cycles of severe intermittent hypoxia resulting from periodic collapse of the upper airway during sleep, is an independent risk factor for insulin resistance (44, 45), and treatment with continuous positive airway pressure (CPAP) reverses several metabolic abnormalities in OSAS patients (46). Therefore, it might be that the severity, pattern, and duration of hypoxia exposure may determine the effects on metabolic and cardiovascular health.

## Conclusion and Future Directions

Adipose tissue dysfunction in obesity is a key factor in the pathophysiology of obesity-related chronic metabolic and cardiovascular diseases. Recent studies have indicated that alterations in AT PO_2_ may drive adipose tissue dysfunction in obesity, although many questions remain. Clearly, more clinical observational and intervention studies in well-phenotyped humans are needed to further investigate AT PO_2_ in obesity, as well as the functional consequences of altered AT PO_2_. Important aspects that need to be taken into account in future studies are the severity, duration, and pattern of PO_2_ exposure, since different experimental conditions may underlie different study outcomes. Moreover, it is of particular interest to examine fat depot-differences (e.g., lower-body versus upper-body) in AT PO_2_, and to unravel whether metabolic and inflammatory responses to chronic physiological O_2_ levels are related to specific fat depots, cell types [e.g., (pre)adipocyte, stromal–vascular cells, macrophages], and/or donor characteristics. In addition, it would be important to understand whether AT PO_2_ relates more strongly to the metabolic phenotype (e.g., insulin resistance) in obesity than to the increased adipose tissue mass itself. Finally, human intervention studies need to be undertaken to determine whether AT PO_2_ can be modified (e.g., weight loss, oxygen therapy) and will subsequently evoke alterations in the metabolic and inflammatory phenotype. These studies should ideally be of integrative nature, combining *in vivo* assessment of adipose tissue and whole-body physiology with measurements in tissue biopsies and cell culture experiments. Studies in this exciting field of research may lead to new opportunities for therapeutic intervention in obese individuals with dysfunctional adipose tissue, thereby improving cardiometabolic health.

## Author Contributions

GG drafted the manuscript. GG and EB revised the manuscript and gave final approval of the manuscript to be published.

## Conflict of Interest Statement

The authors declare that the research was conducted in the absence of any commercial or financial relationships that could be construed as a potential conflict of interest.
